# Inter-cultural and cross-cultural communication through physicians’ lens: perceptions and experiences

**DOI:** 10.5116/ijme.5f19.5749

**Published:** 2020-08-20

**Authors:** Mandana Shirazi, Sari Ponzer, Nazila Zarghi, Fatemeh Keshmiri, Maryam Karbasi Motlagh, Davoud Khorasani Zavareh, Hamid R. Khankeh

**Affiliations:** 1 Department of Clinical Science and Education, Södersjukhuset, Karolinska Institutet, Stockholm, Sweden; 2 Educational Development Center of Tehran University of Medical Sciences, Medical Education Department, Faculty of Medicine, Tehran University of Medical Sciences, Tehran, Iran; 3 Educational Development Center, Sadoughi University of Medical Sciences, Yazd, Iran; 4 School of Public Health and Safety, Shahid Beheshti University of Medical Sciences, Tehran, Iran; 5 Department of Health in Emergency and Disaster, University of Social Welfare and Rehabilitation Sciences, Tehran, Iran

**Keywords:** Doctor-patient communication, physicians, cross-cultural, inter-cultural, Iran, Sweden, Middle East

## Abstract

**Objectives:**

This study aimed to explore Swedish physicians’ perceptions regarding physician-patient communication in an Iranian context and to obtain a deeper understanding of their lived experience when encountering Middle Eastern and Swedish patients in their daily work.

**Methods:**

This is a multi-method study, including conventional content analysis in combination with phenomenological methodology. A triangulation approach to data collection and analysis was used. Serving the purpose of the study, twelve Swedish physicians with previous experience of Middle Eastern patients were purposely selected to participate in the study. They watched a video showing simulated patient encounter in an Iranian context. The video served as a trigger. Semi-structured interviews were conducted focusing on the participants’ perceptions of the video and their lived experiences. Constant comparative analysis was used for a deep understanding of the data.

**Results:**

The core themes were cultural diversity, doctor-centeredness, and patient-centeredness. Cultural diversity was a convergent theme and included trust, interpersonal interaction, context, and doctor dominancy. Patient-centeredness and doctor-centeredness were divergent themes and included doctors’ authority, equity, the experience of illness, and accountability.

**Conclusions:**

The participants confirmed large cultural differences in doctor-patient communication when encountering Iranian and Swedish patients. Inter-cultural and cross-cultural competencies were made visible. To be able to appreciate other cultures’ health values, beliefs, and behaviors, increased cultural competence in health care is of importance.

## Introduction

Cultural competency among health care professionals has become a significant patient safety issue, owing to increasing immigration rates and health problems that are often labeled as “migration-related stresses among immigrants.”^1^ Cultural competency has been defined as an understanding of a culture’s shared beliefs, norms, and values, including their thoughts, styles of communication, ways of interacting, views of roles and relationships, practices, customs, and behaviors regarding these issues.^2^ Culture determines how we explain and value our world, and it provides us with a lens through which we can find meaning. We are all influenced by and belong to multiple cultures that go beyond race and ethnicity.^3^^, ^^4^ In multicultural countries, there is substantial evidence regarding the acquisition of cultural and linguistic competencies among health care providers, as well as among health care organizations.^5^ Furthermore, according to the Accreditation Council for Graduate Medical Education definitions, the core competencies of medical doctors comprise interpersonal communication as well as professionalism. Culture in health care has been defined as “The phenomena to demonstrate sensitivity and responsiveness to patients’ culture, age, gender, and disabilities.”^6^ Cross-cultural competence is understood as a range of cognitive, behavioral, and affective components that enable individuals to adapt effectively, while inter-cultural competence can be viewed as skills that lead to effective and appropriate communication with people of other cultures.

The World Health Organization (WHO), the Association of American Medical Colleges,^7^ and some universities, e.g., Stanford University, have included cultural competence in their medical curriculums.^8^ According to the recommendations of international bodies, different health care systems and health care providers should achieve cultural competency, especially when there is cultural diversity among different nationalities. Owing to these recommendations, physicians should consider the expectations of patients who come from different cultures with dissimilar health care policies (e.g., Western and Middle Eastern countries).

According to Euro Communication II final report,^9^ Sweden provides health services similar to those of other European countries, such as Belgium, Germany, Switzerland, and Spain. On the other hand, Iran, a Middle Eastern country, represents one of the largest minorities in Sweden, amounting to about 1% of the population. Moreover, the immigration rate of Iranian physicians to European countries has grown in recent years.^10^ Thus, familiarity with the largest population of minority groups is beneficial.

Exploring inter-cultural and cross-cultural communication with the use of simulations and standard videos as well as physicians’ reflections has rarely been reported in qualitative studies.^11^^-^^13^ Typically, two main strategies for doctor-patient communication have been acknowledged worldwide: doctor-centeredness or the paternalistic approach, and patient-centeredness. Although patient-centeredness is more common in most Western countries, the paternalistic approach persists, for example in several US states as well as other developed countries such as Japan.^14^ Therefore, it seems that the doctor-patient communication style is an ongoing issue, which needs to be addressed. There is no defined and fully accepted method for doctor-patient communication, even though in most Western countries “patient-centeredness” approach is common.

To our knowledge, only a few studies have explored Swedish doctors’ perceptions and lived experiences (which are the two extremes of one spectrum) regarding communication with Middle Eastern patients in the same study. However, recognizing how to interpret and integrate findings into clinical practice has been addressed only in a few studies.^15^ There is some evidence that perception is regarded equal to attitudes and lived experiences equal to performance.^16,17. ^

This study aimed to explore Swedish physicians’ perceptions and experiences regarding their inter-cultural and cross-cultural communication with Middle Eastern patients. To achieve this aim, we defined three objectives: First, to explore Swedish physicians’ perceptions regarding physician-patient communication in Swedish and an Iranian context. Second, to obtain a deeper understanding of physicians’ lived experience of encountering Swedish and Middle Eastern patients in their daily work, third, to interpret the integrated Swedish physicians’ perceptions and lived experiences into clinical practice by applying a multi-method qualitative approach.

## Methods

### Study design

This is a multi-method qualitative study^18^ in which both conventional content analysis and descriptive phenomenology were used to collect and analyze the data. Accordingly, methodological triangulation for data gathering, and thereby combining data from different sources were used to obtain a comprehensive data set. Initially, conventional content analysis was used to categorize the factors affecting the inter-cultural and cross-cultural communication of the physician with Middle Eastern patients. Following that, in the second step, the phenomenological methodology was used to explore the participants´ lived experiences on interactions with foreign and Swedish patients.

### Participants

The participants were working in outpatient and inpatient services in Stockholm, Sweden. Data collection took around three weeks. Twelve Swedish physicians, nine males and three females (five < 40 years, six between 40–55 years, and one over 70 years of age), who were faculty members and had previous experience with patients from Iran and other Middle Eastern countries (at least 10 patients), and who spoke fluent English, were purposefully selected as key informants. Ten of these physicians had 10–20 years of clinical experience and two had more than 20 years of experience. Four of the participants were orthopedic surgeons, two were pediatricians, and the rest were general practitioners (Table 1).

Ethical approval was issued by the Ethical Committee of Tehran University of Medical Sciences, Faculty of Medicine. According to Swedish law, ethical approval is not required for studies in an educational context. All participants individually signed the informed consent forms regarding their agreement to participate and this study was conducted in accordance with the Declaration of Helsinki.

### Data collection procedure

**Figure 1.**
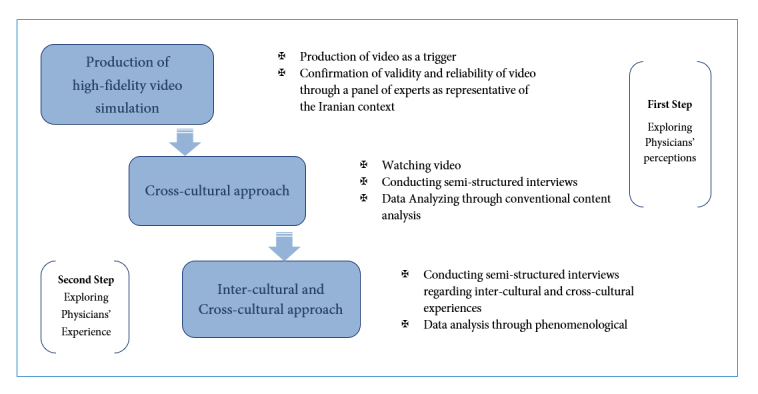
This multi-method qualitative study design consists of two steps, first to explore Physicians’ perceptions using conventional content analysis and second to explore physicians’ experience through phenomenological method

A two-stepped approach was used to explore the communication of physicians’ perceptions and experiences with Middle Eastern patients in two different cultures (Figure 1).

In the first step, physicians watched a 10-minute simulated video that showed a patient encounter in which a fourth-year Iranian medical student and a standardized patient (SP) were assigned to interact. This video, produced in a simulation setting, has been used since 2012 in the Medical Education Department of Tehran University of Medical Sciences (TUMS) for educational purposes.^19^ The communication language in the video was Persian, but it was captioned in English. Two bilingual English experts wrote captions individually, and then the captions were back-translated from English to Persian. In this step, all participants were asked to watch the video focusing on the interaction between patients and physicians, their communication, emotional expression, including eye-contacts. The participants wished to hear the intonation of the subjects’ voices even if they did not understand the Persian language. Iranian experts confirmed the fidelity of SPs’ video.^19^ The content was confirmed by an Iranian expert panel consisting of 10 experts from different disciplines: emergency medicine, psychiatry, medical education, and general surgery. They obtained consensus on both the SPs and students playing their standard role in the Iranian context. This standardized video was representative of the Iranian clinical context and presumed to represent an example of Middle Eastern countries. The simulated encounter on the video was used as a trigger for reflection. The following semi-structured interviews were conducted with a focus on the participants’ perceptions of what they had seen in the video, what they had felt and their insights about encounters with Iranian patients. Moreover, their perception of verbal and non-verbal communication was explored.

**Table 1:** Demographic characteristics of participants (n=12)

Participant	Gender	Work experience	age
P 1	male	10	36
P 2	male	11	39
P 3	female	10	35
P 4	male	15	44
P 5	male	15	46
P 6	female	23	60
P 7	male	14	39
P 8	male	29	77
P 9	male	18	52
P10	female	16	48
P 11	male	12	36
P 12	male	16	53

One of the authors (MS) conducted all interviews that were carried out in English. These recorded interviews were transcribed verbatim (by FK and MK). Constant comparative analysis was applied as a method for qualitative content analysis. These records were analyzed repeatedly and rigorously, paying particular attention to the participants’ dialogues (who says what, when, and how), their embodied behaviors (the relative location, orientation, and movement of the people). Initially, they were asked, “what do you remember of your encounter with patients from Middle Eastern countries?” The interview continued until the saturation of the data occurred. In the cases where there seemed to be ambiguity, a second interview was also conducted. Each interview was listened to several times (data emersion).

In the second step, directly after the initial interview, a second interview was conducted, focusing on the participants’ own lived experience counseling Swedish and Middle Eastern patients employing the phenomenology method. Accordingly, the semi-structured interviews started with general questions went into detail regarding verbal and non-verbal communication with the patients and the reactions that they had seen during their encounter with the Iranian or Middle Eastern patients.

These two interviews with each physician took approximately 70 to 90 minutes and yield 24 units of experiences. The participants received an incentive (two cinema tickets) for taking part in the interviews.

### Data analysis

The data analysis consisted of both conventional content and phenomenological analysis.

The conventional content data analysis was done according to Graneheim and Lundman.^20^ MS analyzed the data under the supervision of HKH and in collaboration with the other authors. To understand the concepts in-depth and avoid superficial and mechanical coding, coding and categorization of concepts were implemented manually by paper and pencil followed by categories and subcategories.

For the phenomenological descriptive data analysis, a descriptive analysis in seven steps, based on the recommended method by Colaizzi^21^ was used. The familiarization step was done by reviewing the Swedish physicians’ accounts several times (emersion). After identifying and condensing meaning units, these were categorized based on their similarity and differences of themes. Following that for formulating meaning relevant to the phenomena, this method was selected to explore the meaning of the Swedish physician’s experience regarding their interaction with the Middle Eastern patients in the clinic. Each interview was transcribed verbatim in English, also all physicians’ thoughts, feelings and ideas were written as data. At this stage, important phrases and statements in relation to the research questions were highlighted and extracted from each interview and coded. Overall, 458 codes were extracted at this stage. This process was checked through an expert’s panel with the research team until the team reached consensus. At the next stage, the meaning units, related to the research question, were chosen out of the sentences. Then, the meaning formulated from every significant statement was chosen and organized into themes (cluster themes). In this phase, one theme was common in content analysis and phenomenology, and one theme was distinguished in both methods. The themes were used to describe the experience. To describe the phenomenon more in-depth, the data was interpreted into clinical practice to identify physicians’ cultural competency for their patients' encounters (Swedish and Iranian). Then clustered themes and formulated meanings were incorporated into the description to create its overall structure, to ensure that all elements of the experience had been included.

The exhaustive description was reduced to an essential structure of the interaction between Swedish physicians and Iranian patients. The data was also incorporated into clinical practice to identify cultural competence for a better understanding of communication between physicians and patients when they encounter Swedish and Iranian patients.

### Trustworthiness

Credibility was achieved by prolonged engagement with the data (2013–2015), member checking, and peer checking (HKH, SP) and by constant comparative analysis. The attributes of trustworthiness include researcher eligibility (familiarity of both principal investigator and research team members with both cultures studied) and the application of an appropriate research methodology.^22^  Credibility was supported by MS being familiar with Swedish (PhD and affiliation to Karolinska Institutet) and Iranian context (faculty member in medical education at TUMS). Moreover, triangulation with interviews, movie analysis, investigator triangulation, methodological triangulation (phenomenology and content analysis) added to trustworthiness. Frequent debrieﬁng sessions and the authors reﬂective commentary with participants were other methods to increase credibility. The study themes were derived from an inductive qualitative content analysis of participants’ perceptions and experiences, using constant comparative analysis throughout the conceptualization process, including identifying meaning units, coding them, categorizing codes (main concept as category and other concepts as a subcategory), and exploring themes. Dependability was verified via data, methods, theoretical, and investigative triangulations. Memos were taken during the interviews (MS) also supported the data analysis process, since using memos can control biases in the research data. Confirmability was based on expert checking (MS, SP, HKH) and peer checking (FK, MK, NZ). Transferability was ensured via tick description of the method, concept verification, which constituted the major output of conclusions related to inter-cultural and cross-cultural views.

## Results

The results were organized into three parts: the participants’ perceptions, their lived experiences and the integration of both. The following core themes emerged: “Cultural diversity,” “Doctor-centeredness,” and “Patient-centeredness.”

Cultural diversity was the only convergent theme and included four main concepts: trust, interpersonal interaction, context, and doctor dominancy. Trust and interpersonal interaction were also verified across the study (Tables 2 and 3).

**Table 2:** The participants’ perceptions regarding physician–patient communication after watching a video in an Iranian context

Theme	Main concept	Concept	Code	Meaning Unit (A sample of quotation)
Cultural diversity	Trust	Belief*	Religious statement Religious statement now in Iran vs. in Sweden	Quote: Difference, Amen – religious statements during conversation Quote: Religious statement, like 60 years ago in Sweden. Participants 1,2,6,10.
		Ø Patient value* Ø Patient respect Ø Religious*	Importance of considering patient value Exaggeratedly Doctor Respect Wearing hijab and religious manner	Quote: Patients are not convenient during history taking due to exaggerated respect to doctor that might be related to prestige and value. Participants: 2,3,5,6,8,10 Quote: Patient appearance and wearing hijab cloth may be related to mistrust for the doctor. Participants: 2,7,8,9
	Interpersonal interaction	Ø Insufficient communication	Verbal: Lack of greeting Nonverbal: Lack of eye contact and physical touch Lack of physical movement	Quote: Doctor doesn’t look at his patient. He speaks with insufficient tone and pitch to his patient. It is interesting that he doesn’t move when his patient came in. Participants: 1,2,6,8,10 Quote: Doctor did not listen to patient and it seems that he is just asking the list of prepared questions. Participants: 2,4,5,7,8 Quote: when the patient feels nervous and told to doctor that she was worried the doctor did not explain complete why endoscopy is not necessary. Participants: 3,4,5,8,9,10
	Context	Ø Sociocultural situation Ø Doctor ascendency	Keeping distance between doctor and the patient Doctors look at the patient from up to down	Quote: Patient and doctor sit beside each other in Swedish setting, but in this video, they sit in front of each other and table was in between. Participants: 1,2,4,6,8 Quote: In the video showed that doctor looked at the patient from up to down and did not consider her at the same level of himself. Participants: 10, 12
Doctor centeredness	Doctor authority	Doctors confidence	Could not convince patient No final agreement between doctor and patients	Quote: It seems that the patient feels inconvenience during the visit. p Participants: 1,2,3,8, 11 Quote: Doctor couldn’t understand her problem as she explained. Participants:3,5,9,10
		Doctor paternalistic view	Doctor pseudo confidence Patient not convinced	Quote: But he did not consider share decision making so patient was not convinced. Participants 1,2,5,6,10 Quote: If the physicians have weak communication skill to convince the Patient. She will go to another DR to do the endoscopy. Participants: 1,3,4,5,9,12 Quote: Doctor doesn’t justify patient about the decision made about his problem Participants: 3,4,5,7,9
		Doctors socio- cultural class	High status of doctor in social hierarchy effect on doctor’s behavior	Quote: Doctor authority seems to be based on doctor’s position and social class instead of scientific knowledge or clinical skill. Participants: 1,3,5,6 Quote: I think there is no need to self-presentation or show power to patients. Participants: 1,2,5,7,8,10
		Feedback and attention	Doctor does not pay an attention and give feedback	Quote: Doctor either does listen to patient carefully or give them feedback. Participants: 4,5,8,9 Quote: I think he doesn’t pay attention to his patient at all. Participants: 1,2,4,7,8
Cultural diversity	Doctor Dominancy	Ø Patient convinced	Patient not convinced	Quote: I believe if patient who is from another culture do not trust to doctor he or she will not use the doctor's prescription and it will threaten patient safety. Participants: 2,4,5,6,8 When patient does not believe the doctor based on their own culture and mind map then they regret to accept the physician opinion and prescription. Participants: 1,3,4,5,10
		Ø Historical background	Doctor centeredness considered as a dominant strategy	Quote: Doctor centeredness was common in Sweden 60 years ago and among older physicians than current doctors in Sweden. Participants: 8,1
	Interpersonal interaction	Ø Insufficient communication	Verbal: · Shortage of interpreter · Language barrier · Translation Problem Non-verbal: · Body language	Quote: " I remember the Iranian patient who was suffering from femur fracture in ER. I had many difficulties to communicate with her then one of the staff (Iranian) who was around by chance helped me to translate and convenience for having surgery. helped us to save patient life Participants: 1,2,5,10 Quote: Communication with body language, nonverbal and Google translator. Participants: 3,4,5,9,10
	Trust	Ø Believes* Ø Professionalism Ø Patient rights* Ø Preserve patient dignity	Delivering bad news and considering patient rights Lack of breaking bad news to patient, not telling the truth regarding sickness	Quote: We believe that it is normal to give any news related to health problem, first to the patient, but in some cultures, doctors prefer to give especially bad news to others instead to the patient. Participants: 2,4,7,9,10
		Ø Ethics and values Ø Patient appearance Ø Religious* Ø Gender	Considering human being in different culture	Quote: All patients sh­­­ould be treated in the same way because they are given equal right to live, ­­­­­­diagnose and treat. Participants: 1,4,5 Quote: Iranian doctor and patient have some barriers in communication skills such as different gender and they do not have eye contact, these are important barriers – in the Swedish culture it means the one does not pay attention to patient. Participants: 1,2

Note: concept verification marked with*; Participants expressed similar perceptions as the quotes indicate are noted by their participant number below the quotes.

**Table 3:** Swedish doctors’ experiences regarding physician–patient communication (concept verification)

Theme	Main concept	Concept	Code	Meaning Unit (A sample of quotation)
Patient centeredness	Equity	Ø Patient belief* Ø Patient experience Ø Shared decision making	Doctors decision making is depending on patient belief Shared decision making will bring patient convey	Quote: Sometimes I make a decision depending on the patient's culture and mind map. Participants: 1,3,5,7 Quote: Involving patient through decision making will end up to her convince. Participants: 3,5,7
	Illnesses experience	Ø Emotional expression	Different pain expressions due to their different cultures	Quote: As I remember, patients from Middle East express their pains much more severe than Swedish patient's. Participants: 3,4,5,12 Quote: Sometimes eastern patient pain threshold is lower than western patient and it is hard for me to estimate how serious their illness is. Participants: 1,2,6,8
	Doctors accountability	Ø Clarify patient situation Ø Patient satisfaction	Patient satisfaction Being sure of patient receiving message	Quote: I myself describe the situation for patient. Participants: 1,3,5,6 Quote: I prefer to clarify situation for my patient, he should feel satisfaction when he leaves my office. Participants: 1,2,4,8 Quote: I try to understand him to convey message of convenience. Participants: 1,3,5,9

Patient-centeredness and doctor-centeredness were divergent themes (Tables 2 and 3). The main concept of doctor-centeredness was doctors’ authority, and the main concepts of patient-centeredness were equity, the experience of illness, and doctor accountability.

### Perceptions after watching the video

Two themes, cultural diversity and doctor-centeredness emerged from the analysis of the participants’ perceptions after they watched the video. The first theme, cultural diversity, consisted of three main concepts: “trust,” “interpersonal interaction,” and “context.”

The concept of trust was based on patients’ values, religion, beliefs, and expectations, all of which have previously been recognized as significant issues in the relationship between the public and health care professionals.^23^^, ^^24^ Four of the participants mentioned religious aspects during their encounters (Table 2), with one stating:

“There are different religious statements, such as ‘Amen,’ that I heard on the video. This religious statement was like those used in Sweden 60 years ago when patients used religious statements.” (P6, female, age 60)

In the video, the physician’s behavior was understood to be based on social status, which led to exaggerated politeness toward the physician; thus, even if the patient did not agree with the doctor, he or she would not express any dissatisfaction or distrust. Several participants (Table 2) related to this aspect, with one remarking:

“Patients are not convenient during history taking due to exaggerated respect for doctors, which might be related to the prestige and value of doctors.” (P8, male, age 77)

Interpersonal interaction included insufficient communication, such as avoiding eye contact, guarded physical movements, and restrained greetings (Table 1). This finding can be perceived in terms of cultural context: In countries such as Iran, doctors have a higher status in the sociocultural hierarchy. In this regard, one participant said:

“The doctor doesn’t look at his patient. He speaks with insufficient tone and pitch to his patient.” (P1, male, age 36)

The main concept of context emerges from the sociocultural positioning and physician dominancy concepts. It is related to physical distance, which was maintained between the physician and patient:

“The video showed that the doctor looked down on the patient and did not consider her at the same level as himself.” (P10, female, age 48)

The second theme, doctor-centeredness, included the main concept of doctors’ authority and related concepts, such as physicians’ sociocultural class, paternalistic views, confidence, feedback, and attention. Doctor-centeredness or “medical paternalism” was related to feelings and perceptions of authority and a lack of attention toward patients’ needs, preferences, and values, leading to illness-centeredness instead of patient-centeredness during the consultation. For example, the paternalistic view was noticed by several participants (Table 1), with one observing:

“He did not consider shared decision making, and the patient was not convinced. He has high pseudo confidence.” (P10, female, age 48)

### Participants’ own lived experiences

Two themes also arose from the research question regarding the participants’ own lived experiences, namely “cultural diversity” and “patient-centeredness.”

The cultural diversity theme consisted of three main concepts, i.e., doctor dominancy (historical background), interpersonal interaction, and trust. Interpersonal interaction included aspects of insufficient communication. The related codes were as follows: the shortage of interpreters, language barriers, translation problems, and guarded body language (Table 3). Several mentioned language barriers, with one noting:

"I remember an Iranian patient who was suffering from femur fracture in the ER. I had many difficulties to communicate with her until one of the staff (Iranian) who was around by chance helped me to translate and convince the patient to have surgery. This staff helped us to save the patient’s life.” (P5, male, age 46)

The theme of patient-centeredness had three main concepts: equity, the experience of illness, and doctor accountability. The patient-centered approach helped physicians to see the experience of illness through the patients’ lens. The patient also assisted by seeing the disease from the doctor’s point of view, which helped them both to reach a shared decision about diagnosis and treatment. The concept of the illness experience, defined as emotional and pain expressions due to cultural differences, was mentioned by several, with one stating:

“As I remember, patients from the Middle East express their pains more severely than Swedish patients.” (P3, female, age 35)

The memos showed that one of the older physicians (P8, male, age 77) had a divergent view compared to those of the others. He perceived no difference between the Iranian clinical setting and the Swedish one. He said: “Swedish and Iranian settings show no differences from each other.” Three younger physicians, on the other hand, pointed out that historically Sweden had a more doctor-centered approach, but

**Table 4:** The interpretation of results to fill the existing research gap through an integrated approach

Integrated themes	Themes	Perceptions (Attitudes)			Lived experiences (Performance)		
		Main Concept	Concept	Codes	Main Concept	Concept	Codes
Convergent	Cultural diversity	Trust	Belief	Religious statement at the present time in Sweden vs. Iran	Trust	Delivering bad news as a form of professionalism and patient rights Lack of giving direct Breaking bad news to patient Do not tell the truth regarding sickness	Beliefs Professionalism Patient rights Preserve patient dignity
			Patient value Patient respect Religious respect	Importance of considering patient value Exaggeratedly doctor respect Wearing hijab and religious manner		Considering human beings in a different culture	Ethics and values Patient appearance Religious Gender
		Inter- personal interaction	Inappropriate communication	Verbal: Lack of greeting Nonverbal: Lack of eye contact and physical touch Lack of physical movement	Interpersonal interaction	Verbal: Language barrier Shortage of interpreter Common language barrier Nonverbal: Body language	Inappropriate communication
		Context	Sociocultural situation	Distance between the doctor and the patient, a table placed between them	Doctor Dominancy	Patient not convinced Doctor centeredness considered as a dominant strategy about 60 years ago in Sweden	Patient convinced Historical background

that patient-centeredness has been an evolving trend during the past few decades. One of these physicians noted:

“Doctor-centeredness was common in Sweden 60 years ago, and even nowadays it is still more common among older physicians than the younger ones.” (P2, male, age 39)

In order to provide a more comprehensive picture of the Swedish physicians’ perceptions and experiences, we applied an integrated process for the convergent and divergent themes. The interpretation of these results and their implications for clinical practice are shown in Table 4.

## Discussion

In this study, we used an integrated approach to merge data related to participants’ perceptions and experiences, through which the themes emerged and were verified. The participants, Swedish physicians, were asked to narrate how they felt about, perceived, and experienced communication with patients from another cultural context compared to their own. Perceptions were translated to attitudes and to experiences to represent a feasible knowledge translation to operational terminology as also used in other published studies. The overlapping themes (convergence) of their attitudes and their experiences (previous performances) regarding communication with their Middle Eastern patients were noticed. Moreover, divergent themes were also identified. These themes arose when the participants’ perceptions of communication in an Iranian context differed from their own lived experience, i.e., inter-cultural and cross-cultural differences were noticed.

### The convergent themes

Cultural diversity was the major overlapping and common theme. Cross-cultural communication involves both verbal and nonverbal communication. The propensity to evaluate behavior from other cultures as either appropriate or insufficient depends mostly on one’s own cultural preference.^25^ In the current study, the results regarding verbal and nonverbal communication as concepts and codes were related to these aspects.

Trust emerged as an important and common main concept and seemed to be one of the factors that most influenced the views of health care providers working in the Swedish health care system. Aligned with our study, another inter-cultural qualitative study conducted in Sweden, entitled “Experiences of the Swedish Health Care System: An Interview Study with Refugees in Need of Long-Term Health Care,”^26^ focused on contact with health care providers. Two main concepts, “Care organizations/resources” and “Professional competence,” emerged in their study. In terms of professional competence, the authors stated that if physicians do not communicate well with their patients, then the patients will not trust them and will doubt their professional competence.^26^ Consistent with our study, another study conducted in the Netherlands^27^ explored a health care delivery system in a multicultural society and suggested five strategies for adapting to cultural diversity, two of which focused on planning to develop better communication between health care providers and patients. Further, in multicultural contexts, providers with a wide range of capabilities, such as trust and a high degree of self-reflection, could be achieved through robust training.^27^^-^^29^ Therefore, policymakers must be aware of the need for education in cultural competency, not the least for ensuring the safety of patients from other cultural backgrounds.

Another main concept underpinning cultural diversity was interpersonal interaction, including verbal and nonverbal communication, such as limited eye contact and different body language. These cultural perspectives were clearly emphasized by the participants. It is known that in some Middle Eastern countries, eye contact is an improper behavior if the physician and the patient are of opposite genders, which is why verbal communication is more important and effective in these contexts. On the other hand, in Western countries, if the physician does not look directly into the patient’s eyes, it would be considered ineffective communication and would indicate that the doctor is not paying enough attention to his/her patient.^14^ Thus, in order to establish an effective relationship between Western doctors and Middle Eastern patients, a range of aspects of cultural diversity needs to be taken into consideration.

Context will also influence the mode of communication between patients and health care providers.  In a systematic review by Rocque and Leanza, the effect of culture, micro-culture, and contextual issues on communication in medical settings were demonstrated.^30^ In our study, the Swedish participants found the Iranian context, with a table placed between the doctor and the patient, as contributing to the patient’s mistrust and indicating a doctor-dominant approach. It seems, then, that Swedish doctors should be aware of the Iranian or Middle Eastern countries’ contexts and consider the various types of issues that might affect their foreign patients’ trust.

Claramita and colleagues^31^ found in their studies in South Asia, that “Social distance” and “Closeness of relationships” were important. They stated, “Doctors unintentionally adhere to behavior that underlines social distance, while patients seem dissatisfied with that type of behavior and doctors unintentionally fail to acknowledge or notice (it).” Moreover, they noticed that the desk was placed between the doctor and the patient, and that when the doctor greeted the patient, he stood up, but he did not move toward the patient in the clinical settings. This finding, similar to our findings, can be interpreted as a paternalistic doctor situation.^31^^,^^32^ However, it should be noted that there are different patterns of communication even among Asian countries. For example, studies done in Indonesia suggest that nonverbal cues are used more frequently than verbal communication: Patients do not ask for more information, but nonverbal communication, such as eye contact, is frequently used.^31^^,^^32^ Therefore, it is of great importance to be aware of the cultural differences between countries, even those within the same region.

### The divergent themes

The main distinction regarding the divergent themes was doctor-centeredness and patient-centeredness. The participants’ perceptions and their clinical experiences were based on their daily work, where patient-centeredness is the established model in Swedish health care. In a study by Dahm on patient-centered care strategies, they explored international medical graduates’ communication skills during their encounters with simulated patients in a communication skills course in Australia.^15^ Their findings were in line with ours in terms of the application of a paternalistic approach by the Iranian physicians. The main difference between their study and ours was the methods used. We interviewed Swedish doctors regarding physician-patient communication after they had watched a video from an Iranian setting, whereas Dahm assigned a standardized patient from the other context, showing this video encounter to students and asking them to explain their experiences regarding the “challenges of patient-centered care.”^15^ However, despite the different methods employed, the results were similar.

Our results are also in line with the findings by Kalengayi and colleagues.^33^ They carried out a qualitative study on Swedish health care providers’ perceptions and experiences with immigrant patients to identify the dilemmas they face and producing knowledge appropriate to the formation of policies and clinical practices. It is noteworthy that they emphasized that several issues at different levels (i.e., macro, exo, meso, and micro) influence clinical practice, and that Swedish health care policies (macro-level) and clinical practices within the health care system (exo, institute) are patient-centered. They also found that immigrant doctors made clinical decisions by themselves without considering the patients’ views.^34^

There is solid evidence, as shown in this and related studies, that language acts as a barrier to effective communication between health care providers and immigrant populations.^34^^-^^36^ Our findings suggest that language barriers are an obstacle to accurate interpersonal interaction, significantly inhibiting communication and thereby impeding the elicitation of necessary data from foreign patients, in turn threatening the patient-centered approach.

Patient-centeredness was also one of the main themes about which participants believed that they already related with their patients via a patient-centered strategy, i.e., inter-cultural perspective.^36^^-^^38^ However, based on their observation of the video, they reflected on the fact that consultations were more doctor-centered and that their Middle Eastern patients seemed to prefer doctor-centeredness, especially when it came to newcomers. Therefore, the participants thought that the transition from a doctor-centered to a more patient-centered approach should take place gradually.

One of the participants, the oldest among them, found no difference between Iranian and Swedish physicians’ communication experiences. The source for his divergent perception might be related to his graduation year and to the change in physician-patient communication strategies in subsequent years, i.e., the historical background that emerged from the interviews and the memos. Hence, it is expected that the Iranian doctor-centered strategy will gradually change into a patient-centered approach over time.

### Methodological aspects

In this study, we aimed to address both inter-cultural and cross-cultural approaches. Claramita and colleagues^32^ and Rosenberg and colleagues^39^ studied physicians’ communication competencies, using an inter-cultural approach. The latter used a video produced from real family physicians’ encounters with patients with mental disorders from another culture. The family physicians were interviewed after watching their own videos, i.e., inter-cultural reflection. Others, for example, Kleinman,^40^ emphasized cross-cultural perceptions in health care communication. However, in our study, we used a simulated video from another culture and asked the participants to reflect on both the video and their own lived experience, which in turn allowed us to apply a dual approach, i.e., to explore the participants’ perception and experiences regarding communication skills as seen from both cross-cultural and inter-cultural views.

To the best of our knowledge, there are no existing publications focusing on physician-patient communication between Iranian patients and Swedish physicians in which cultural differences are addressed through their perceptions and experiences in everyday practice. For the third objective, we interpreted the data for clinical practice, considering the physicians’ perception as attitude and physicians’ lived experience as performance. The concepts, obtained from this study, could be considered as a core of cultural competency (such as a trust) for reforming health care providers’ curricula in pre- and postgraduate degrees.

In our study, the video was used as a trigger to inspire the physicians to express their thoughts during the interview and to encourage them to recall their previous experiences. It also gave the physicians the opportunity to directly observe clinical consultations in another context. Overall, using qualitative multi-methods (conventional content analysis and phenomenology method plus triangulation method), we used a novel data collection and analysis process that would potentially improve understanding of the phenomena under study. Moreover, few qualitative studies have applied a simulated method and standard videos as a trigger for stimulating participants to reflect on both the video and their previous experiences regarding communication with Iranian patients.^11^^-^^13^

The memos used in this study were helpful but also raised new questions, among them being the view of the older participant, who, after watching the video, saw no differences between Swedish and Iranian physician-patient communication. One of the authors (MS) contacted some of the other participants regarding this contradictory view for clarifying and re-checking the results. The participants reconfirmed their view that the video of the Iranian context depicted a doctor-centered approach that was once common historically in Sweden. Hence, for the future physicians’ education in Iran, it might be beneficial to shift from a doctor-centered to a patient-centered approach.

### Limitations

One limitation of this study, although the simulated video was intended to be representative of the Iranian clinical context, was undoubtedly, the fact that the presence of a physician in a real context might have led to better interpretations. The small sample size might also be considered another limitation, even though in this qualitative study, data saturation was reached through data richness from several sources and via data triangulation.^24^  Further, our results though cannot be generalized, and we do believe they contribute to cross-cultural and inter-cultural knowledge about the physician-patient relationship in general as well as in Sweden and quite likely in some other European countries as well.

## Conclusions

The participants of this study confirmed the large cultural differences in doctor-patient communication when encountering Iranian and Swedish patients. Inter-cultural and cross-cultural competencies were made visible to underline the fact that all health care professionals should be aware of and sensitive to patients' needs and beliefs. To achieve this goal, it is necessary to reform health care providers' curricula to include cultural competency courses. This modification should enable health care providers to appreciate other cultural values, beliefs, and behaviors, which is of paramount importance. Further studies are needed to investigate if a change in educational practices will have an impact on future inter-cultural and cross-cultural clinical work.

### Conflict of Interest

The authors declare that they have no conflict of interest.

### Acknowledgements

Special thanks to the Swedish physician participants for their valuable contributions to this study and to the Iranian medical students who played the simulation video which was used as a trigger in this study.
